# Comprehensive Approaches of Nanoparticles for Growth Performance and Health Benefits in Poultry: An Update on the Current Scenario

**DOI:** 10.1155/2022/9539908

**Published:** 2022-09-17

**Authors:** Ilyas Ahmad, Zia-Ur-Rehman Mashwani, Naveed Iqbal Raja, Abeer Kazmi, Abdul Wahab, Amir Ali, Zohaib Younas, Saman Yaqoob, Mehdi Rahimi

**Affiliations:** ^1^Department of Botany, PMAS-Arid Agriculture University Rawalpindi, Pakistan; ^2^Institute of Hydrobiology, Chinese Academy of Science, University of Chinese Academy of Sciences (UCAS), Wuhan, China; ^3^Shanghai Center for Plant Stress Biology, CAS Center for Excellence in Molecular Plant Sciences, Chinese Academy of Sciences, Shanghai 200032, China; ^4^Department of Biotechnology, Institute of Science and High Technology and Environmental Sciences, Graduate University of Advanced Technology, Kerman, Iran

## Abstract

Currently, providing nutritious food to all people is one of the greatest challenges due to rapid human population growth. The global poultry industry is a part of the agrifood sector playing an essential role in food insecurity by providing nutritious meat and egg sources. However, limited meat production with less nutritional value is not fulfilling the higher market demands worldwide. Researchers are focusing on nanobiotechnology by employing phytosynthesized mineral nanomaterials to improve the growth performance and nutritional status of broilers as these mineral nanoparticles are usually absorbed in greater amounts from the gastrointestinal tract and exert enhanced biological effects in the target tissues of animals with greater tissue accumulation. These mineral nanoparticles are efficiently absorbed through the gastrointestinal tract and reach essential organs via blood. As a result, it enhances growth performance and nutritional value with less toxicity and tremendous bioavailability properties. In this review, the research work conducted in the recent past, on the different aspects of nanotechnology including supplementation of mineral nanoparticle in diet and their potential role in the poultry industry, has been concisely discussed.

## 1. Introduction

Nanotechnology is the promising and emerging technology that has tremendous potential to revolutionize livestock and agriculture sectors. The concept of nanotechnology was to reduce the size of particles to few nanometers. The field of nanotechnology is not only applicable to basic sciences research but is also performing a major role in disease diagnosis and therapeutic agents. Nanotechnology is an advent grade technology that is aimed at creating materials in which at least one dimension is less than 100 nm [1–5]. Nanoparticles are not new world creation; historically, nanoparticles existed hundreds of years ago, as many natural phenomena on the planet earth like forest fires, volcanic eruptions, and photochemical smog result in a formation from these nanoparticles [1]. Nanotechnology is concerned with the phenomenon of converting larger-sized molecules to a particle as small as nanosize [6, 7], while converting these larger compounds to smaller ones results in a modified chemical and physical properties of the base material. These changes include changes in the degree of solubility, absorption of the particle, transportation across the cell and tissues, and excretion, and also, the most important is antagonism (Mohapatra, Swain, Mishra, Behera, Swain, [8, 9]). In the modern scientific world, nanoparticles are almost used in every field of science like in the field of agriculture. Nanofertilizers are used to improve crop production and also used to remove toxic substances and water catchers, and nanoparticles are also used to eradicate certain pollutants from the water and air; the cosmetics industry is also now loaded with the products containing nanoparticles majorly used in skin care and also in dyes. One of the most promising applications of nanoparticles is in the human medicine where it has enabled the scientist for controlled liberation of cancer drugs, hormones, nutrients, and gene therapy [10–13]. Recent studies have also showed that nanominerals are more potent and they are more easily available for cells as compared to their larger sized materials [14].

Minerals are important for animals because they are required to carry out various physiological and biochemical reactions in the body of organisms, for normal growth and to continue their race for the future [15, 16]. Many feeds given to animals and especially to poultry have a very low level of minerals, and if they are available in enough amount, their bioavailability is very low, but on the other hand, the animal requires mineral supplements in larger quantity because along with the use of mineral at a cellular level, a significant amount of minerals is excreted to the environment which results in increased cost [17, 18]. The bioavailability of the nutrients does not depend upon the particle size and its potency, but it is also influenced by the physiological rate, species, source, and synthetic structure of the components [19]. Nanofeed substances not only increase feed proficiency but also reduce the cost of feed along with additional benefit of improved yield in terms of animal products [20].

## 2. Types of Nanoparticles

Organic, inorganic, dispersions, emulsions, and nanoclays are the different types of nanoparticles that are classified based on their chemical properties. Inorganic nanoparticles are used extensively in feed products like titanium dioxide which act as a feed colorant and are used as an ultraviolet barrier in feed packaging. Minerals are also used in feeds as well as in the packaging industry, which involves nanoclays in the packaging of feed; other minerals like silver, magnesium, and calcium are being used as antimicrobial agents, as water purifiers, and in feed storage. Fat, sugar, and protein molecules are classified as organic nanoparticles. Organic nanoparticles by altering feed and its bioavailability also improve their nutritional value. Organic nanoparticles can encapsulate nutrients and can transport them through the bloodstream, which is now referred to as the nanocapsule. Due to increased bioavailability, nanocapsules are used to deliver the nutrients without altering the taste and appearance. These encapsulated nanomaterials are incorporated into feed as liposomes and also as a biosensor in the feed packaging system, shelf-life extender, identification markers, and antimicrobial agents in stored feed. Nanoemulsion is another class of organic nanoparticles that is mainly used as a stabilizing agent, to deliver the active components either in the water/oil interface or in a continuous phase [21].

### 2.1. Synthesis of Nanoparticles

**Figure 1:**
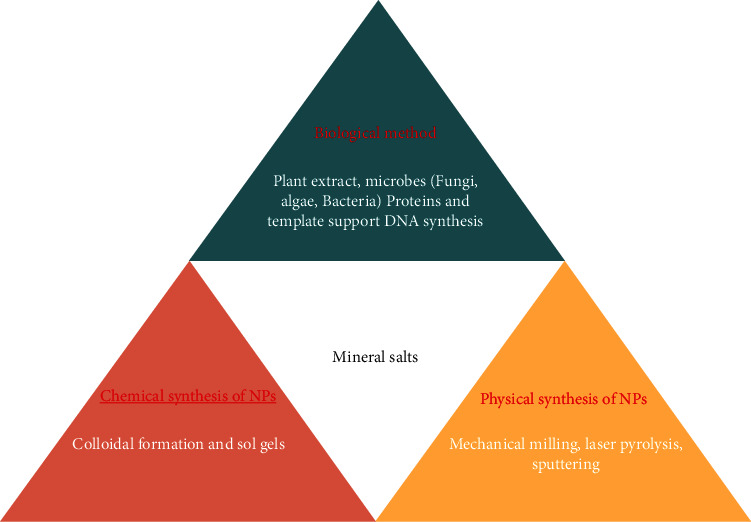
Biological, chemical, and physical methods of nanoparticle synthesis.

The synthesis of nanoparticles mainly depends upon the need and the purpose for which they are intended to be used. The stability of the active component, the toxicity of the nanoparticle, its liberation, and its possible effect on the living system are also taken into account when they are intended to be used for living systems. Nanoparticle synthesis can be broadly classified into chemical and biological methods (Figure 1). As the ultimate fate of nanoparticles in one way or the other is a human being, the chemically synthesized nanoparticles are not considered safe as compared to phytosynthesized nanoparticles ([22]: [23]).

## 3. Biological Method

In this method, mainly microbes, algae, fungi, and plant extract with active molecular compounds and other biological agents are used [22–24]. Green nanotechnology is the term used for the synthesis, characterization, and assessment of the biological effect of phytosynthesized nanoparticles such as silver, copper, gold, iron, zinc, and selenium on living systems. The plant extract has a major role as it acts as a reducing and capping agent for the synthesis of nanoparticles [25–28].

**Figure 2:**
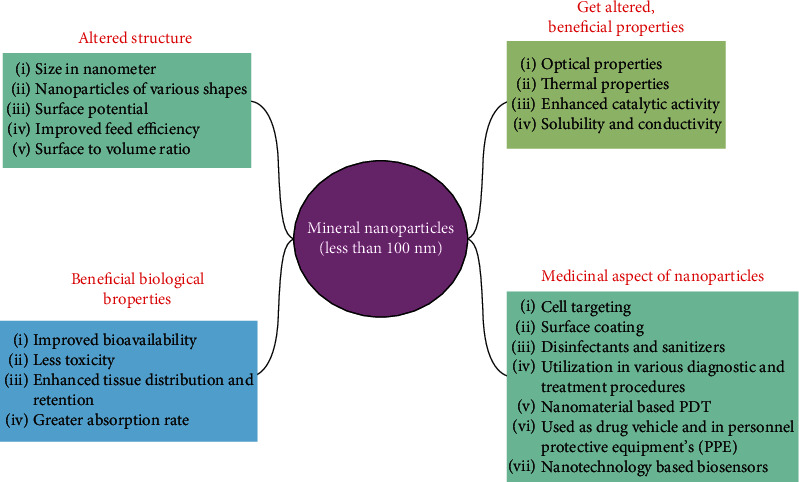
Altered and improved features of mineral nanoparticles with their beneficial aspects for different applications.

In addition to the quality and safety of foods, nanotechnology has emerged as a technological advancement that can transform agriculture and the food sector which will enhance global food production with high nutritional value and better quality with the safety of food as well [29, 30]. Nanoparticles and the organic molecules found in food, i.e., carbohydrates, proteins, and lipids, work at the same scale. A metal oxide nanoparticle, i.e., titanium dioxide, is one of the most used nanoparticles for various industrial and commercial purposes and also in the food industry [31] (Figure 2). Silver nanoparticles due to their antimicrobial activities are one of the most widely used nanoparticles in the food sector [32].

The antimicrobial activity of silver nanoparticles has been proved in various studies, in which AgNP has shown antimicrobial activity against a wide range of microbes such as gram-positive and gram-negative bacteria, yeast, mold, and viruses.

Salmonellosis and campylobacteriosis are the two most frequently reported food-borne pathogens caused by Salmonella and Campylobacter. These two pathogens are of the highest concern in the poultry field, which are present either in the gut content or in the skin of birds which may become part of the human food by using poultry meat [33, 34]. Fortunately, both microbial species are heat sensitive and their transfer can be prevented by adequately cooking the meat [33]. The globally median number of 5% of illnesses and 16% of deaths due to food-borne pathogens is caused by *Campylobacter* species. A major share of this food-borne pathogen can be attributed to chicken meat which acts as a food vehicle for these infectious agents [35]. Contamination is not only limited to farms; it can be caused at any step from farm to kitchen. Now, the industry is following the “farm to fork” philosophy to emphasize all parties for the prevention of contamination. The process starts with the breeding stocks and continues through hatcheries, farms, feed mills, live-poultry pickup and transport, processing plants, distribution channels, and the consumer's own kitchen. Nanotechnology offers several benefits which can be exploited during different phases of the food chain, which can be utilized to improve microbial quality of food during production, processing, transport, and storage. Commercial poultry processing environment also plays a major role in reducing the chance of food-borne contamination, prior to consumer supply and use.

## 4. Nanotechnology and Poultry

**Figure 3:**
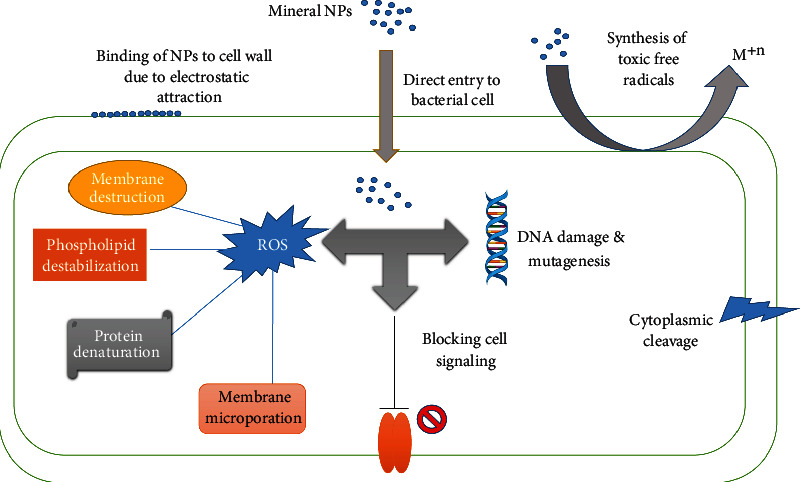
Potential role of chromium nanoparticles to improve various growth parameters and enhanced immunity in poultry.

Many reports are available in the literature about the utilization of nanoparticles in poultry either in feeding, watering, or also through other routes to improve bird's health (Table 1). The antimicrobial and immunomodulatory effects of many nanoparticles including gold, silver, and titanium are well-established. Silver nanoparticles cause immunostimulation and antimicrobial activity in the animal. [36–39]. The use of chromium nanoparticles significantly improves the thyroid production, growth, weight of liver, semen quality, egg size, feed conversion ratio, and shell thickness [40–42] (Figure 3).

Use of antibiotics in poultry feed to increase poultry production is not new, as different concentrations of antibiotics have been used varying with growth, time, and breed of chicken. Due to rapid microbial resistance to antibiotics, these drugs are now banned in various countries. So there is a dire need to use alternatives, which should benefit for poultry growth and health performance with less harm to humans. The importance of nanotechnology in improving meat and egg production has been greatly neglected, mainly due to lack of sufficient knowledge and limited literature [43]. Nanoparticles of different minerals are the best alternatives which have better penetrance, more absorption, and greater conversion ratio which make them a very good candidate to be used instead of these antibiotics. In the following discussion, we will discuss about the use and benefits provided by various mineral nanoparticles for poultry growth and production [44].

### 4.1. Silver Nanoparticles (AgNPs)

**Figure 4:**
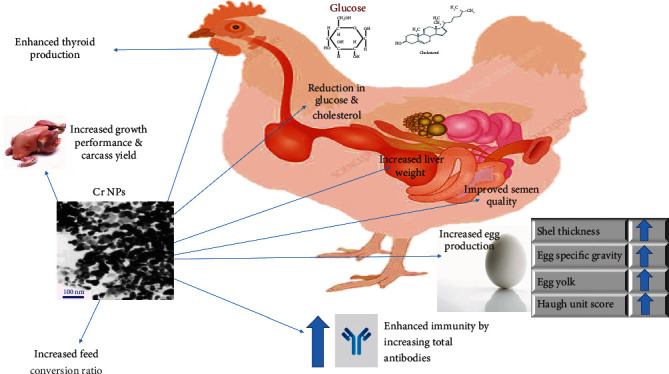
Mode of action of mineral nanoparticles, by entering the cell either directly or indirectly, causes genotoxicity, blocking the signaling pathway, or may cause destruction of major organic compounds.

Silver nanoparticles have been proved effective not only for gram-positive and gram-negative pathogenic strains of bacteria, but it has also shown antibacterial activity against resistant bacterial strains [45, 46]. Silver nanoparticles have also shown an antibacterial property against *Staphylococcus aureus* and *Escherichia coli*, by degrading the cell wall at minimum inhibitory concentration of 50 and 100 ppm, respectively [47]. As far as the mode of action of nanoparticles is concerned, NPs attach to infectious and pathogenic microbes and eventually remove them from the bird's body (Figure 4). According to [48], silver nanoparticles at a dose of 4 ppm/kg resulted in reduced growth of *E. coli*, without affecting the population of beneficial bacteria, like lactobacillus species in microflora. Silver nanoparticles at a dose rate of 900 ppm increased the body weight, feed intake efficiency, and feed conversion ratio ultimately on overall growth performance in birds ([49] in nutritional). It is recently being observed that silver nanoparticles are now being used also in solution and suspensions as well. Owing to their well-established and confirmed antimicrobial properties, silver nanoparticles are now being widely used as disinfectants and also to reduce the metabolic wastes like nitrogen oxides and ammonia. Dobrzanski et al. (2010) demonstrated in their study that nano-Ag has biocidal activities which has not only reduced the number of harmful *E. coli*, salmonella, and streptococcus species, but also, it reduced the total number of bacteria.

It is proved by various research studies that silver nanoparticles stimulate inflammatory signaling by producing reactive oxygen species (ROS), which results in activated macrophage cell thereby secreting tumor necrotic factor type alpha (TNF-*α*). This increased concentration of TNF-*α* results in membrane damage and ultimately cell death. If silver nanoparticles are recognized as foreign particles by the immune cells, it may also result in multistep and multilevel immune response which finally may lead to toxicity [50]. However, somehow, if the immune system is unable to recognize AgNPs, then the capacity of silver nanoparticles to activate the response of immune system decides their fate in the host, in spite of all this *in vivo* studies that have confirmed that nanoparticles are also responsible for promoting inflammation [51]. A study conducted by

Grodzik and Sawosz [52] assessed that silver nanoparticles at a concentration of 10 ppm reduce the size and number of follicles but had no major effect on the growth performance of the chickens [53] administrated at different doses (20, 40, and 60 ppm) of silver nanoparticles to assess its effect on bursa, which revealed that the increasing dose of nanoparticles affected the number of follicles. It may be concluded that silver nanoparticles may have effected and reduced the microflora of the gut due to its antimicrobial properties, as silver nanoparticles carry the available oxygen, which reduces the growth of strictly anaerobic bacteria, which has manifested itself by reducing the growth of bursa of Fabricius. Almost no effect of AgNPs was reported on Immunoglobulin G (IgG) and Immunoglobulin M (IgM) levels ([37]. But it has also been showed that silver nanoparticles in combination with certain amino acids like threonine and cysteine can improve the innate and adaptive immunity in chickens during embryonic development [39, 54].

### 4.2. Zinc Oxide Nanoparticles

Zinc is one of the most vital trace metals which is required to initiate and complete most of the essential pathways and to carry out various physiological functions [16]. Zinc is required by all the six major types of enzymes and the total number of enzymes estimated to be 200, which are associated with the metabolic pathways of major organic compound like carbohydrates, proteins, lipids, and nucleic acids. Moreover, zinc also plays a major role in various signaling pathways usually by initiating a response with hormone secretion pathways as well. The immune defense system also requires zinc at different levels either for immune response initiation or in various intermediate regulatory steps in the immune response pathway (S. [15]). Like many other minerals, zinc also functions as a cofactor for various enzymes in the liver, including alanine aminotransferase, gamma glutamyl transferase, and aspartate aminotransferase. Deficiency of zinc reduces the activity of these enzymes which leads to various liver diseases including hepatitis and cirrhosis [55]. Zinc also makes an integral part of antioxidant enzyme system, especially superoxide dismutase, which is one of the most important players of the organisms' body against oxidative stress. Therefore, deficiency of zinc also affects the activity and capacity of the antioxidant defense system [16].

Bioavailability of organic zinc as compared to inorganic is higher, but one major problem associated with organic zinc is its high cost [56]. Moreover, higher usage of zinc in dietary supplements also results in higher rate of excretion which causes environmental pollution [57, 58]. Furthermore, high concentration of zinc also affects the stability of other nutrients, such as vitamins as well, which necessitates that zinc should be used at an optimum level. Additionally, it is also observed that long-term exposure also causes deposition of zinc in the animal body, as zinc residue has been reported in various organisms living in a high zinc environment [59, 60].

Nanotechnology has been extensively used in the last few years in the field of animal husbandry and nutrition, mainly to improve the utilization of various trace minerals and to improve their bioavailability [61, 62]. Zinc is also important for poultry as it is required for the maintenance of growth performance, for the development of skeleton, and in immune response system. It is also added in various poultry diets at a rate of 0.12 to 0.18 g/kg, mainly for the improved development of bone and feathers and to enhance the immune response (S. [15]). Zinc sulfate and zinc oxides are the two major sources of inorganic zinc which is used at commercial level in poultry feed, out of which 80-90% zinc is used as ZnO which has very low bioavailability as compared to ZnSO_4_ [63]. On the other hand, zinc sulphate is not safe as it is very reactive which promotes the formation of free radicals, which in turn facilitates various degradative reactions for vitamins, fats, and oils, decreasing the nutritional value of diet [64]. Many studies have been carried out to explore the effect of ZnO nanoparticles to promote growth performance and health status in poultry. [65] used zinc oxide nanoparticles of 40 nm size at a dose rate of 30, 60, 90, and 120 mg/kg in basal diet of broilers that has improved the feed intake, feed efficiency, and weight gain of broilers in first 21 days at 60 and 90 mg/kg, but higher concentration (120 mg/g) resulted in reduced growth performance and weight loss. (Y. J. C. N. [66]) conducted their study to investigate the comparative effect of large ZnO particles (60 mg/g) and ZnO Nanoparticles (20, 60, and 100 mg/g) fed to broiler chickens. It was concluded that chickens fed with 20 and 60 mg/g of ZnO NPs had low feed conversion efficiency but increased body weight gain as compared to ZnO particles and ZnO NPs at a dose rate of 100 mg/g. According to [8], ZnO NPs at the 1/500^th^ level of basal diet increase overall growth rate, along with increased levels of alkaline phosphatase activity and serum glucose concentration, with decreased activity of alanine aminotransferase enzyme activity. It has been reported previously that ZnO NPs at a supplementation rate of 10, 20, and 40 mg/g in basal diet at about 15°C–18°C induce ascites and increased weight gain. However, lower feed efficiency was seen in birds fed with 40 mg/g of the ZnO NP group. However, there are also some reports that ZnO NPs at a dose rate of 25 and 50 mg/g and ZnO particles at 100 mg/g have negligible effect on feed efficiency, feed intake, and body weight gain and carcass yield. But higher doses of ZnO NPs has reduced malondialdehyde content along with cooking loss of chicken meat as compared to ZnO at 100 mg/g [67].

### 4.3. Selenium Nanoparticles

**Figure 5:**
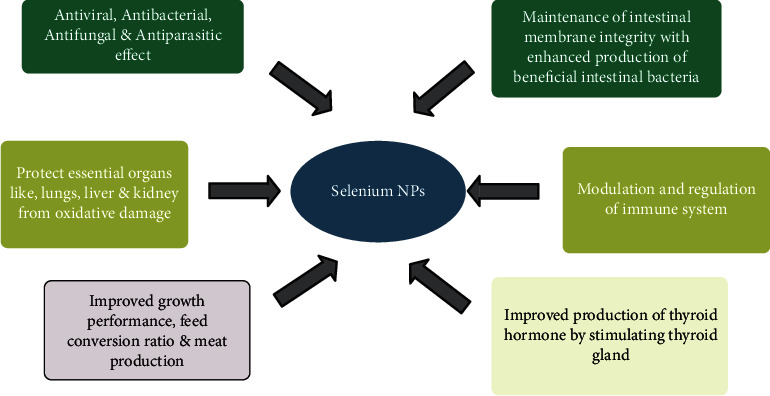
Potential role of selenium nanoparticles in poultry nutrition and their ameliorative effect.

Selenium is also one of the trace elements, required for animal nutrition as it exerts multiple actions related to animal production, fertility, and disease prevention (kryukov et al., 2003). Selenium requirements for poultry ranges from 0.1 to 0.15 mg/kg, which can be supplemented with any poultry feed. Common selenium supplements in various poultry feeds include sodium selenite, inorganic sodium selenite, organic selenomethionine, and selenium yeast. Comparatively, organic forms of selenium are better absorbed in the gastrointestinal tract, compared to inorganic ones. More than 90% selenium in organic form is absorbed compared with 60% absorption of inorganic selenite. Moreover, organic forms such as selenomethionine can be retained in the tissues more efficiently than selenate or selenite [68]. Selenium is the integral part of about 25 selenoproteins which play a pivotal role in enzymatic redox reactions at the cellular level, which enables them to scavenge the reactive oxygen species (ROS). There are many important physiological processes like reduction of oxidised proteins and membranes; biosynthesis of nucleotides; metabolism of hormones, specifically thyroid hormone; and scavenging of toxic peroxides along with transport and storage of selenium reservoir in different tissues (Papp et al., 2007) (Figure 5).

These selenoproteins can be grouped into three categories, glutathione peroxidases (GSH-Px) thioredoxin reductases (TrxR), and iodothyronine deiodinases, out of which thioredoxin reductases are of most importance. This system is responsible for many essential cellular processes including synthesis of DNA (Holmgren 1985, 1989), integral part of body defense system against oxidative species, and maintaining the structure and integrity of the endoplasmic reticulum (Rhee et al., 2005). Moreover, the most important and pivotal role of this system is to convert the inactive form of Thyroxin (T4) to active form (T3), which affects the overall metabolism of the body. Thioredoxin system is also involved in expression of gene regulation through activating various transcription factors like, NF-ƙB, Ref-1, AP-1, P53, glucocorticoid receptor, and apoptosis-regulating kinase (ASK1). So it can be concluded that this system either directly or indirectly is involved in many key process of the body ranging from affecting cell cycle, apoptosis, gene expression, and immune response (Rundlöf and Arnér 2004). The second most important group is GSH-Px that is primarily required for regulating the concentration of free radicles at a cellular level (Back 2013; Sarkar et al., 2015). It has been concluded by different studies that selenium is not directly associated with enhancing growth performance of the poultry; rather, it is indirectly involved in various key process by activating and proper functioning of redox systems, especially TrxR and GSH-Px (Gangadoo et al., 2016).

Two different forms of selenium, inorganic (selnite or selenite) or organic (selenomethionine), are used in avian industry to fulfill the requirement of selenium but both of these have their own disadvantages. The first thing to note is that it is quite a reactive compound that can be reduced by various nutrients, including ascorbic acid, and some feed ingredients to an unavailable metallic form. Feed moisture can also dissolve it and convert it into volatile compounds that are lost. Additionally, sodium selenite has prooxidant properties in a dose-dependent manner, which can negatively affect the gut of animals/chickens. Last but not least, sodium selenite is poor at transferring selenium from eggs to the foetus via the placenta, so the body cannot build reserves of selenium for use during stressful conditions when selenium demands rise while feed consumption usually decreases (Sarkar et al., 2015). In order to overcome these disadvantages and to fulfil the need of “precise nutrition,” the concept of “nanoselenium” has been introduced. Unlike the bulk selenium in the form of sodium selenite, this nanosized selenium possesses many key properties; like it has large surface area, greater intestinal absorption, and higher mucosal permeability.

Moreover, the role of selenium nanoparticles is not only restricted to redox reactions, but it is also involved in promoting epithelial health which leads to enhanced absorption and digestion of nutrients in the intestine. It has been shown that supplementation of selenium nanoparticles in poultry feed at 0.9 mg/kg has shown improvement in population of beneficial bacteria like *Lactobacillus* and *Faecalibacterium* spp., along with production of short-chain fatty acids (SCFas), most importantly butyric acid (Gangadoo et al., 2018). Both of these improvements are important for poultry, as improving gut microbiota enhances gut health and integrity while the SCFas serve as potential energy-rich source for intestinal cells. Moreover, it is also been shown that selenium nanoparticles result in increased population of *Faecalibacterium prausnitzii*, which is not only in excess to normal level but it was also higher than the normal probiotic supplement (Gangadoo et al., 2018).

Supplementation of Se NP at 0.5 mg/kg diet in laying hens improved the rate of egg production, GPx activity, and total antioxidant status in addition to significantly decreasing the soft-shelled or cracked egg rate. Chicken fed diets containing 0, 0.1, 0.3, and 0.5 mg/kg of Se NP improved final body weight, daily body weight gain, and feed conversion ratio after 90 days (X. [69]). Selenium not only plays important role in meat production, but it has also been reported that it can enhance the egg-laying ability of hens as well. Se NP at a supplementation rate of 0.5 mg/kg increased the selenium content in tissues. Selenium is also reported to have a positive effect on the liver, breast muscle, pancreas, and feathers [70]. Selenium supplementation has also shown to have a stimulatory effect on the immune system, improving growth and reproductive performance and enhancing disease resistance. Consequently, deficiency of selenium in poultry diet manifests itself in the form of exudative diathesis, pancreatic dystrophy, myopathy, immunodeficiency, and nutritional muscular dystrophy [71]. From the above discussions, it is apparent that supplementation of Se NP has variable responses compared with the bulk Se sources on production performance of poultry.

### 4.4. Copper Nanoparticles

Copper (Cu) is a vital trace element involved in various physiological and biochemical processes. However, lower absorption of copper in animals is causing the major problem, as most of the copper is excreted, contaminating the environment. Copper has long been used in the poultry diet to improve growth performance and carcass yield. First and the foremost beneficial aspect is its antimicrobial effect, as copper reduces the population of harmful bacteria in broiler intestine which indirectly promotes growth process in chickens. Secondly, copper acts as cofactor for various enzymes; most important are antioxidant enzymes like, GSH-Pox, CuZn-SOD, and intestinal lipase (Mroczek-Sosnowska et al., 2013). Lastly, copper is also involved in stimulation and secretion of various growth hormones which ultimately results in high yield. Copper is also an integral part of enzyme systems which are involved in iron metabolism, formation of red blood cells, and inducing immune stimulation and its proper functioning. Furthermore, it is also been proved by various studies that copper is involved in the formation of connective tissues and enhancing the nervous system [72].

In the last decade, immense work has been done in which mineral nanoparticles were added to the poultry diet which has given many beneficial results. As there are many important physiological and biochemical functions performed by Cu, copper sulphate is added to the poultry diet [73, 74]. It is suggested that the Cu concentration should be 4 mg/kg for layers and 8 mg/kg for broilers [75]. But practically, in the poultry farm, these guidelines are not followed and excessive amount of Cu is added to get more carcass yield. As there are numerous reports which suggest that excessive copper has toxic effect, majority of the reports concluded that Cu can become toxic if it is added 100 times the recommended dose. Moreover, these higher doses of copper result in resistant bacterial population in the chickens. These antibiotic-resistant bacteria ultimately reach to humans and cause various health problems. Therefore, there is a need of a time to give attention to “precise nutrition,” in which accurate amount of minerals is added to the feed, which benefits the poultry as well as the humans.

One of the most suitable alternatives to copper sulphate (feed additive in poultry) is CuO nanoparticles which have greater potency, more absorption, and better interaction with other organic and inorganic materials due to their smaller size and large surface area. The Cu-NP has the capability to cross the small intestine and distribute into the blood, brain, heart, kidney, spleen, liver, and intestine (Montero et al., 2017). There are many studies which suggest the antibacterial effects of Cu-NPs on strains mainly *E. coli* and *Staphylococcus* spp. A study demonstrated that the antimicrobial properties of chitosan implanted with Cu-NP reduce gut bacteria such as *E. coli*, Enterococcus faecalis, S. aureus, and, particularly, Lactobacillus fermentum, which is one of the primary targets of antibiotic growth promoters, suggesting that the Cu-NP could be used to minimise undesirable levels of microbial populations without causing cytotoxicity [76] Further, Cu-NP inhibits the growth of *S. aureus*, *B. subtilis*, *E. coli* bacteria, *Micrococcus luteus*, *Klebsiella pneumonia*, and *Pseudomonas aeruginosa*. The subsistence rate of *E. coli* and *B. subtilis* bacteria is decreased by increasing Cu-NP concentrations [77].

### 4.5. Gold NPs

Gold and its compounds with multiple applications in medical field have long been utilized in for drug delivery, drug targeting, etc. Gold NPs due to their no or zero cytotoxicity, large surface area, and other biocompatible properties have long been used in nanobiotechnology and nanomedicine [78]. Au NPs can reach to the GI (gastrointestinal tract) through watering and feeding and also can be given via oral or through injection of therapeutic nondrug. Because of the small size of the particle, it fastly diffuses GI tract mucous and ultimately reaches to the blood circulation and intestinal cell lining [79]. As the Au NPs are diffused through the GI tract, they (Au NPs of 100 nm or less) can also translocate through lymphatic to various important organs such as the spleen and liver. Au NPs of smaller size like >50 nm are more potent and are capable of to be taken up by the villus epithelium [80]. Au NPs when entering into the cell also have the ability to upregulate certain genes as well. It also enhances growth of the breast muscle and improved and enhanced protein synthesis with increase in the population of beneficial bacterial.

## 5. Conclusion and Future Perspectives

Rapid and uncontrolled increase in human population requires enhanced agriculture production to meet the food requirement. The field of animal nutrition is facing immense pressure to increase production to meet the growing demand of animal protein. To meet the egg and meat demands, most of the farmers use antibiotics in poultry feed which not only enhances the yield but also prevents infectious diseases in chickens. Lab-made antibiotics such as tetracycline are now extensively used in poultry feed in most parts of the worlds. This is positive side of the picture; yes, there is negative side as well. This extensive use of antibiotics causes threat to the human population either by reducing the microbiota in human gut or conferring antibiotic resistance to many pathogens as well. Scientists are now working on some natural alternatives to antibiotic so that the egg and meat production should be appropriate to meet the market demand with less or zero toxic effects. Nanobiotechnology is a tremendous field with potential application in animal and especially in poultry with increasing applications in diagnostics, medication, and nutrition. The concept of nanobiotechnology is based on the fact that by changing the particle size to nanometer, it makes these particle more potent, increases bioavailability, and increases retention in the body. Nanoparticles are synthesized mainly by three methods, chemical, physical, and biological. Biological synthesis or phytosynthesis of nanoparticles is recently getting more attention as this process involves plant extract (usually medicinal plants) as a reducing agent, the phenomenon also known as “green nanotechnology.” Various mineral nanoparticles mainly of copper, iron, zinc, titanium, selenium, and silver have been used for animal and mainly for poultry nutrition. Many studies have been carried out to investigate the actual mode of action of nanoparticles, which have contradictory results as far as the growth improvement is concerned. As these are small-sized particles, they are efficiently absorbed through the GI tract and by reaching essential organs and blood, they exert immense biological effects on target tissues. These mineral nanoparticles result in increased carcass yield, growth performance, egg laying ability, less toxicity, and improved distribution and bioavailability. Moreover, certain nanoparticles like AgNPs and ZnNPs also have a role to control the risk of food-borne pathogen *Campylobacter*, along with increase in the population of beneficial gut microflora. Some of the studies have also suggested that nanoparticle feeding has also improved immunity, digestibility, and growth performance in broilers.

## Figures and Tables

**Table 1:** A summary of the studies on different nanoparticles (NP) on performances, immunity, and other health effects on poultry.

Nanoparticles	Health effects on poultry	Reference
Zinc nanoparticles	(i) Increased tibia ash Zn content (ii) Increased breast muscle Zn content (iii) Body weight and feed conversion ratio was unaffected	[81]
Zinc nanoparticles	(i) All the egg quality traits and mean egg weight were significantly increased	(Amem & Al-Daraji, 2011)
Copper and zinc nanoparticles	(i) Zinc was accumulated in the liver of broiler chickens (ii) Reduced MDA content (iii) Increased feed consumption and body weight	[82]
Zinc nanoparticles	(i) High doses showed significant changes specially in the liver, congested blood vessels, and proliferation of bile duct	[83]
Carbon nanoparticles	(i) No significant difference was observed in RBC morphology, weight of organs, and other biochemical parameters among the tested and control groups (ii) It was concluded that carbon nanoparticles remain in the body without affecting any major trait	[84]
TiO_2_ nanoparticles	(i) TiO_2_ nanoparticles affected mRNA levels of different genes which are involved in Wnt signaling (ii) Treatment with TiO_2_ resulted in free radical production which disrupted the somite myogenesis and lateral plate mesoderm	[85]
Silver nanoparticles	(i) Increased phagocytosis and leukocyte metabolic activity by application of silver nanoparticles (ii) Antioxidant activity was enhanced with decreasing level of haemoglobin (iii) Increased lipid peroxidation and bilirubin content	[7]
Silver nanoparticles	(i) Silver nanoparticle accumulation was observed in the liver and intestine, and this accumulation was dose-dependent, i.e., higher dose resulted and greater accumulation (ii) Silver nanoparticles resulted in decreased villus height to crypt depth ratio in the jejunum (iii) Stimulated and activated immune with enhanced oxidative stress system was observed in the AgNP-treated group as compared to control	[86]
Silver nanoparticles	(i) Enhanced immunostimulatory effect was observed (ii) Elevated level of IL-6 demonstrated that higher dose of silver nanoparticles has proinflammatory effect (iii) AgNPs also stimulated B (iv) They also stimulated B lymphocytes which resulted in a higher level of immunoglobulins	[86–88]
Selenium nanoparticles	(i) Diet supplementation with selenium resulted in a higher concentration of selenium in different tissues as compared to nontreated groups (ii) It was also demonstrated that selenium source (sodium selenite, nanoselenium, or Se yeast A) had no effect on tissue selenium retention and no significant difference was observed between these groups	[89]
Selenium nanoparticles	(i) Feeding nano-Se increased glutathione peroxidase mRNA expression in the liver (ii) Expression of cytokine genes was also stimulated by feeding with nanoselenium	[90]
Selenium nanoparticles	(i) Improved average daily gain (ADG) and survival ratio (ii) Tissue accumulation of selenium was improved	[91]
Calcium nanoparticles	(i) Greater improvement was observed in average daily gain (ADG), and about 12% improvement was observed in feed conversion ratio (FCR)	[92]
Chromium nanoparticles	(i) Increase in food intake was observed in stressed quills, but no significant difference was observed in nonstressed quills	[41]
Chromium nanoparticles	(i) Cr NP uptake by the apical membrane was reduced (ii) The efficiency of epithelial transport across Caco-2 monolayers was increased using Cr NP	[42]
Chromium nanoparticles	(i) Eggshell thickness and eggshell weight were increased, along with yolk weight, Haugh unit, and albumen height and weight	[40]
Manganese nanoparticles	(i) There was no significant effect on carcass yield	[93]
Manganese nanoparticles	(i) Lower FCR, but no effect was noted in average daily gain (ADG) and feed intake (ii) Blood/liver lipid peroxidation, SOD, and GPx are not affected	[87]
Manganese nanoparticles	(i) Increased tibial bone weight	[94]
